# The Diseases and Disorders of the Ox
*"The Diseases and Disorders of the Ox, with some account of the Diseases of the Sheep," by George Gresswell; and "Additions on Human and Comparative Pathology," by Dr. Albert Gresswell. (London: W. H. Allen and Co., 1889.)


**Published:** 1890-05-24

**Authors:** 


### THE DISEASES AND DISORDERS OF THE OX.*

This is a popular book on the ox, especially with regard to bovine medicine, and, we should imagine, occupies such a place in veterinary science as the various "domestic medicines" do in human medicine. It is well written and fairly up to date, but it is spoilt by too much diffuseness and preachiness. In a book for use and practice we want to go straight to the point, not to wander off into the bye-paths of religious and philosophical disquisition. The Hendon cases of cow disease and their bearing on scarlet fever are fully noted. There is a fair account of the varieties of variola and the theories and practice of vaccination. The use of antiseptics is insisted on, but not enough. There ought to be, as far as possible, as much care taken to prevent germ disease in operations on animals as on man. We know it is confidently stated by some that domestic animals are not so susceptible to bacterial influences as that "superior animal" man is, but we are thoroughly sceptical as to this alleged immunity. Quite rightly, this book insists on the antiseptic plan with regard to parturient sheep. We remember a plague of puerperal septiæmia in a valuable lambing flock which was stayed by moving the remaining ewes, which had to lamb down into a distant field, and making the shepherd purify his hands and arms with carbolic acid. In this case it was noted that whenever the ewe required aid, she invariably died in a few days after from fever, though those which, after moving, required the same assistance, remained healthy. The same shepherd attended both sets of ewes, only latterly he was disinfected. We made many *post-mortems* on those ewes, and they presented much the same appearances as are met with in the puerperal fever of women, while, as in the human cases, there is an intense power of poisoning existing in the corpse, which makes the shepherd very careful in "flawing," or skinning the sheep which have died from this disease. With regard to this point, the book should insist, over and over again, in the obstetrical operations on the cow depicted, that the operator ought to scrupulously cleanse his hands and arms with perchloride solution or other proper disinfectant before introducing his hand into the passages. We have known cases of puerperal fever in the cow produced by the want of these elementary principles of hygiene.

The chapter on anatomy is fragmentary rather than elementary; in a second edition this should be corrected. On the whole, we can commend the book as being an honest attempt to place before the public the state of knowledge with regard to the pathology of the ox in its relation to that of man, and also as a handy book of reference for the treatment of our bovine dependents.

* "The Diseases and Disorders of the Ox, with some account of the Diseases of the Sheep," by George Gresswell; and "Additions on Human and Comparative Pathology," by Dr. Albert Gresswell. (London: W. H. Allen and Co., 1889.)

